# Migration stopover ecology of Cinnamon Teal in western North America

**DOI:** 10.1002/ece3.8115

**Published:** 2021-09-21

**Authors:** Desmond A. Mackell, Michael L. Casazza, Cory T. Overton, J. Patrick Donnelly, David Olson, Fiona McDuie, Joshua T. Ackerman, John M. Eadie

**Affiliations:** ^1^ U.S. Geological Survey Western Ecological Research Center Dixon CA USA; ^2^ Intermountain West Joint Venture – U.S. Fish and Wildlife Service Missoula MT USA; ^3^ U.S. Fish and Wildlife Service Division of Migratory Birds Denver CO USA; ^4^ Department of Wildlife, Fish, and Conservation Biology University of California Davis CA USA

**Keywords:** avian migration, GPS telemetry, habitat selection, spectral mixture analysis, waterfowl, wetland conservation

## Abstract

Identifying migration routes and fall stopover sites of Cinnamon Teal (*Spatula cyanoptera* septentrionalium) can provide a spatial guide to management and conservation efforts, and address vulnerabilities in wetland networks that support migratory waterbirds. Using high spatiotemporal resolution GPS‐GSM transmitters, we analyzed 61 fall migration tracks across western North America during our three‐year study (2017–2019). We marked Cinnamon Teal primarily during spring/summer in important breeding and molting regions across seven states (California, Oregon, Washington, Idaho, Utah, Colorado, and Nevada). We assessed fall migration routes and timing, detected 186 fall stopover sites, and identified specific North American ecoregions where sites were located. We classified underlying land cover for each stopover site and measured habitat selection for 12 land cover types within each ecoregion. Cinnamon Teal selected a variety of flooded habitats including natural, riparian, tidal, and managed wetlands; wet agriculture (including irrigation ditches, flooded fields, and stock ponds); wastewater sites; and golf and urban ponds. Wet agriculture was the most used habitat type (29.8% of stopover locations), and over 72% of stopover locations were on private land. Relatively scarce habitats such as wastewater ponds, tidal marsh, and golf and urban ponds were highly selected in specific ecoregions. In contrast, dry non‐habitat across all ecoregions, and dry agriculture in the Cold Deserts and Mediterranean California ecoregions, was consistently avoided. Resources used by Cinnamon Teal often reflected wetland availability across the west and emphasize their adaptability to dynamic resource conditions in arid landscapes. Our results provide much needed information on spatial and temporal resource use by Cinnamon Teal during migration and indicate important wetland habitats for migrating waterfowl in the western United States.

## Graphical abstract

We analyzed 61 fall migration tracks of fall migrating Cinnamon Teal in western North America. Furthermore, we investigated habitat selection patterns at fall stopover sites. Our results can be used to further inform waterfowl conservation and management efforts in the United States and Mexico.

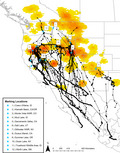

## 1 INTRODUCTION

With over half of the world's migratory birds experiencing population declines, the need to protect these species across all life‐history stages, including migration, is apparent (Runge et al., 2015). Waterfowl in particular rely on continental wetland networks supporting migratory pathways that connect important breeding and wintering grounds (Haig et al., 1998; Johnsgard, 2010). Globally, 30% to 90% of these networks are threatened or have been heavily modified or destroyed by human development (Abramovitz, 1996; Brophy et al., 2019; Moser et al., 1996). From the 1780s to the 1980s, the United States lost approximately 50% of its wetlands with some states experiencing even more extreme losses. For example, California lost more than 90% of its historic wetlands during this period (Dahl, 1990). The sustainable use of wetland resources is essential to maintain a balance between wildlife needs and socio‐economic stability (Loiselle et al., 2001), but the increasing prevalence of droughts in a system transitioning to a more arid climate means that habitat managers operate with limited water supplies (Seager et al., 2007; Wang et al., 2018; Williams, Cook, et al., 2020).

Fall‐migrating Cinnamon Teal traverse much of the semi‐arid regions of the southwestern United States where wetland loss has been extensive and water is limited (Dahl, 1990; Gammonley, 2020). Water regimes in this region are variable, both temporally and spatially, which may make migratory birds in this region more opportunistic and less committed to local sites (Robinson & Warnock, 1997; Skagen et al., 2005). This also results in phenological misalignment between waterfowl migration timing and annual wetland flooding patterns (Donnelly et al., 2019). Since survival and fecundity in fall‐migrating dabbling ducks can be associated with the chronology and duration of time spent at stopover sites (O’Neal et al., 2012), it is essential to understand habitat selection patterns during migration and how waterfowl adapt to a dynamic landscape (Kasahara et al., 2020; Palm et al., 2015). This information is critical for effective population management and habitat conservation of migratory species that require management across different local, state, and national boundaries (Chevallier et al., 2011; Hutto, 2000; Palm et al., 2015).

Cinnamon Teal are one of the least studied dabbling duck species in North America. The breeding range of the North American subspecies extends from southern Canada, across the western United States and Mexico, with major breeding grounds in the Intermountain West and Central Valley of California (Gammonley, 2020). Data on distribution and abundance are lacking because Cinnamon Teal populations often occur in areas that are not covered by traditional waterfowl surveys (e.g., Breeding Bird Survey, Midwinter Survey) and, even when surveyed, Cinnamon Teal are combined with Blue‐winged Teal, limiting the survey's utility for Cinnamon Teal (Baldassarre, 2014; Sauer et al., 2017). In addition, waterbirds in arid regions, such as the Great Basin, have not been surveyed well due to the vastness and inaccessibility of the region (Warnock et al., 1998). Furthermore, information on Cinnamon Teal migration and habitat use along migratory routes is limited. Our comprehensive study tracking movement and space use along migration routes augments traditional surveys to better understand the distribution of the species and potential threats they face.

In this study, we examined the migration chronology and space use of Cinnamon Teal along fall migration routes in western North America. We focused on the fall migratory period because this is one of the driest periods of the year and wetland availability is often limited in the arid and semi‐arid regions traversed by migrating Cinnamon Teal (Donnelly et al., 2019; Gammonley, 2020). Migration chronology and habitat use patterns were derived by analyzing 61 fall migration tracks from birds marked with GPS‐GSM transmitters over three years (2017–2019). We overlayed these stopover locations with remotely sensed habitat landscape data in which we classified the underlying land cover into 12 habitat classes (Table 1). By gathering locations of Cinnamon Teal movements across the western United States and Mexico, we were able to measure habitat selection at stopover sites as well as identify the variance in habitat selection among ecoregions (Cold Deserts, Great Plains, Mediterranean California, Southern Semi‐arid Highlands, Tropical Forests, and Warm Deserts).

**TABLE 1:** Descriptions of each habitat classification used in our analysis

Habitat Classification	Description
Dry agriculture	Agriculture that is not flooded. Includes both active and fallow fields.
Dry nonhabitat	Areas not classified as wet in the spectral mixture analysis or categorized as dry agriculture were grouped as dry nonhabitat. Often associated with undeveloped uplands, shrubland, forests, and urban zones.
Golf and urban	Ponds in golf courses and urban developments.
Lake and reservoir	Large naturally occurring lakes and damned reservoirs. Does not include agricultural and urban ponds.
Managed wetland	Actively managed wetlands with clear dikes where water levels are controlled. Includes wetlands on public wildlife areas and private land such as duck hunting clubs.
Natural wetland	Naturally occurring wetlands outside the floodplain of riparian systems and not delineated by artificial dikes or levees (e.g., Playa Lakes).
Ocean	Open ocean. Includes the Pacific Ocean as well as the Gulf of California.
Riparian	Include both large rivers like the Rio Grande as well as small riparian systems and the floodplains associated with them.
Shrimp farm	Classification exclusive to the shrimp farms that occur along the coast in Mexico.
Tidal	Tidal estuaries and shallow flats.
Wastewater	Includes wastewater ponds from sewage treatment facilities, feedlots, mines, refineries, metal works, power plants, and other industrial operations.
Wet agriculture	Wetlands associated with agriculture. Includes stock ponds, small impoundments, irrigated ditches, and flooded fields.

## 2 METHODS

### 2.1 Study area

**FIGURE 1:**
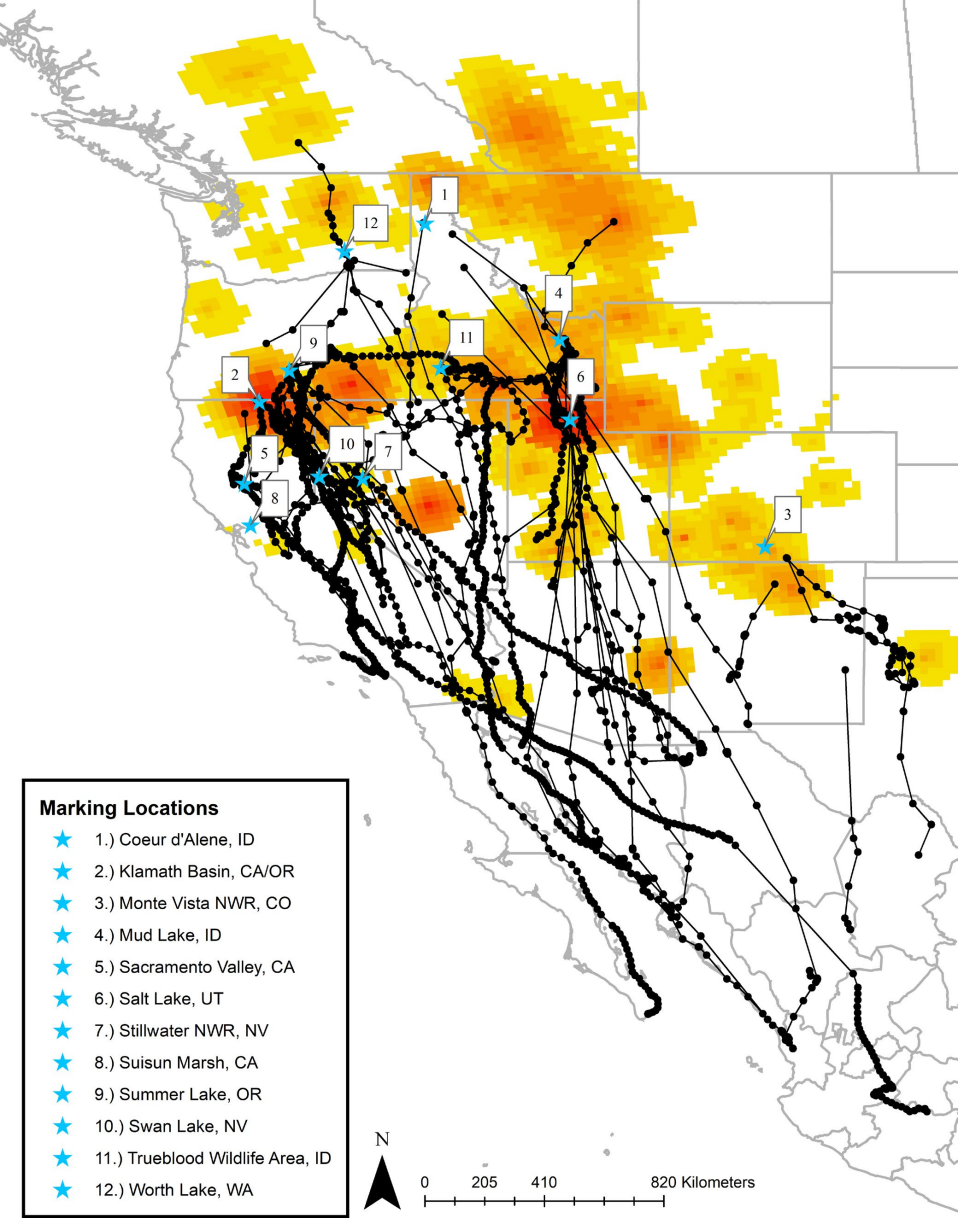
Geographic locations where deployment of GSM‐GPS tracking devices on Cinnamon Teal occurred across seven states during the years of our comprehensive tracking study (2017–2019). Black points and lines represent fall migration locations and tracks for the 61 birds analyzed in our study. Locations are overlaid against summer abundance maps (2011–2015) provided by the North American Breeding Bird Survey (Sauer et al., 2017)

Cinnamon Teal were marked in the spring and summer to coincide with banding efforts already in place at important breeding and molting areas in seven different states encompassing most of the breeding range in the United States (California, Oregon, Washington, Idaho, Utah, Colorado, and Nevada). Specific marking locations include the Suisun Marsh, CA (38.143° N, 121.974° W); Sacramento National Wildlife Refuge Complex, CA (39.427° N, 122.165° W); Summer Lake, OR (42.927° N, 120.776° W); Lower Klamath National Wildlife Refuge, CA and OR (41.956° N, 121.707° W); Great Salt Lake, UT (41.411° N, 112.113° W); Mud Lake/Camas National Wildlife Refuge, ID (43.887° N, 112.435° W); Coeur d’Alene River Wildlife Management Area, ID (47.463° N, 116.586° W); Trueblood Wildlife Area, ID (43.008° N, 116.108° W); Worth Lake, WA (46.608° N, 119.077° W); San Luis Valley, CO (37.488° N, 106.102° W); Swan Lake, NV (39.651° N, 119.85° W); and Stillwater, NV (39.587° N, 118.509° W) (Figure 1). Birds were tracked along their fall migration routes and stopover sites across the western United States and through central and western Mexico.

### 2.2 Capture and transmitter attachment

We captured and fit 223 Cinnamon Teal with backpack‐mounted GPS‐GSM transmitters from April 2017 through October 2019. Most birds were captured during the breeding or molting period, and capture method varied based on timing and bird life‐history stage. Just before and after the breeding period, baited funnel traps were used to capture both males and females. Nesting females were captured on nests using a combination of dip nets, walk‐in funnel traps (Dietz et al., 1994), and bow nets placed around the nest (Salyer, 1962). Flightless birds were captured during the molting period using dip nets from airboats. Finally, Cinnamon Teal were captured using rocket nets prior to the hunting season but only within California. Each bird was fitted with a solar‐powered GPS‐GSM tracker (Ecotone^®^ Crex series transmitter (14 g) or Ornitela^®^ OrniTrack‐10 transmitter (10 g)) attached via a backpack harness (Dwyer, 1972). The harness material used was a 9.525 mm wide nylon‐coated neoprene ribbon (Conrad‐Jarvis, Corp., Pawtucket, RI) that was elastic to compensate fit with seasonal physiological changes (Parejo et al., 2021). Transmitters were always <5% of the bird's body mass and were often under 3%. Each device was set to collect locations from every 15 min to every 6 hr depending on battery charge (Appendix S1: Figure A1.1). Data were transmitted to the tag manufacturer using 2G or 3G cellular networks for web‐based retrieval. This study was approved by the U.S. Geological Survey Western Ecological Research Center Animal Care and Use Committee and was conducted under federal banding permit #21142 and state scientific collecting permit #SC‐8090.

Of the 223 Cinnamon Teal marked, 61 provided no data during the fall migratory periods extending from August through December. An additional 11 individuals were excluded due to mortality or hardware failure occurring within 5 days of marking. Because the Central Valley of California contains a large resident breeding population of Cinnamon Teal (Baldassarre, 2014), 43 individuals that remained only in the Central Valley and demonstrated no migratory movements were also excluded from our migration analysis. We were unable to determine the fate of a further 35 birds which were necessarily excluded from the analysis due to insufficient/no migration data. These birds either died prior to migrating, never migrated from their summer range, or no data were obtained due to hardware failure or the bird never moving within cellular range. Finally, 12 individuals were harvested during the hunting season from within their summer range prior to initiating migration and were also removed from the analysis, leaving 61 individuals that provided enough data to be analyzed. We analyzed stopovers for 56 of these birds and migration timing for 60 birds.

### 2.3 Identification of migratory period

To identify and define each bird's migration, we first filtered all locations to include only those between 1 August and 31 December of each year (the fall migration period between summer breeding/molting and arrival to the winter range). We used the first location during August as a point of origin, representing the summer grounds. We then calculated the net displacement (km) for each bird from its origin point to every location along the rest of the track using the adehabitatLT package in R version 4.0.0 (Calenge, 2006; R Core Team, 2020). Localized movements within core summer areas for our birds fell within a maximum net displacement distance of 50 km from the point of origin and migration onset was defined as the moment each bird left this distance threshold without returning. However, we also required birds to ultimately have a net displacement of greater than 150 km from their summer grounds because we observed two individuals making large and recursive exploratory movements up to 148 km from their summer range. These birds both returned to their core summer areas shortly after making these recursive flights. This allowed us to avoid mistakenly categorizing recursive premigratory flights as migratory for birds with incomplete tracks and to separate exploratory summer movements from migratory behavior. We identified the timing of departure as occurring midway between the last stationary summer location and the first migratory movement (Miller et al., 2005). The relationship between the date of migration onset and the latitude of departure was also investigated using linear regression in R version 4.0.0 (R Core Team, 2020). We defined the end of migration as the southern terminus of a bird's migratory route where no further large movements were made (>50 km). We were able to determine complete fall migration for 19 of the 61 birds. It was not possible to determine the end of migration for the remaining 42 individuals due to transmitter failure and mortalities (Appendix S2: Table A2.1), so we used the last known alive location as the end of the track.

### 2.4 Identification of stopover locations for habitat analysis

**FIGURE 2:**
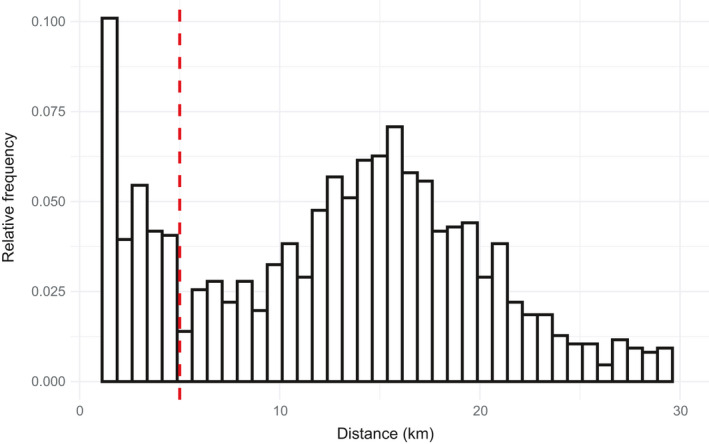
Histogram showing the distribution of the minimum 2‐day nearest neighbor distance for all GPS locations obtained from 61 fall migrating Cinnamon Teal tracked across a 3‐year tracking study (2017–2019). The clear bimodal distribution indicated a behavioral change at 5 km (indicated by the red dashed line). This is the distance at which we separated the two different migration movement behaviors of Cinnamon Teal. All movements below 5 km were classified as short‐distance stopover movements and those longer than 5 km as long‐distance migratory movements

Nonmigratory movements of waterfowl contain two common movement patterns, local scale movements within the same habitat patch, for example, as individuals rest and forage, and recursive movements to prior resting or foraging locations (McDuie, Casazza, Overton, et al., 2019). Migration, by contrast, consists of large‐scale nonrecursive movements. Therefore, it is possible to differentiate migratory from nonmigratory movements by assessing the nearest distance to a point among a set of closely occurring relocations. We discriminated stopover locations from the migration path of each bird by first calculating the nearest neighbor distance to each point occurring within a two‐day sliding window along each animal's track. This produced a distinct separation in the distribution of the “nearest” neighboring relocation occurring at a 5 km distance, which we interpreted as the greatest dispersion of any individual within a stopover location (Figure 2). Therefore, we categorized all locations with at least one neighboring location occurring within 5 km and within two days as a nonmigratory stopover location. Locations with no neighboring point collected within 5 km in a two‐day window were classified as migratory. To classify habitats used within each stopover, we identified and excluded locations where birds were most likely flying (>10 km/hr) by calculating speeds from the location and timestamp data. We used a maximum movement speed of 10 km/hr because median local flight speeds for Cinnamon Teal have been estimated at 36.5 km/hr (Hedenström & Alerstam, 1995; McDuie, Casazza, Keiter, et al., 2019), ducks are said to swim or walk no faster than approximately 5 km/hr (Usherwood et al., 2008) and, if flight does not incorporate the entire duration between GPS locations, flight speed is underestimated. Additionally, we required a bird to have at least three nonflight locations and be in the same spot for at least three hours to be categorized as a stopover location. Finally, because we defined 5 km as the greatest dispersion of any individual within a nonmigrating/stopover location, we buffered each use point within a stopover by 5 km to define our stopover sites and serve as a boundary for available habitat in our selection analysis. We identified 186 stopover sites using this method.

### 2.5 Classification of stopover wetlands

Following methods described by Donnelly et al. (2020), we measured areas of wetland flooding within the 5 km buffered regions around all identified stopover locations. Surface water was estimated using spectral mixture analysis (SMA; Adams & Gillespie, 2006) derived from 30 × 30 m pixel resolution Landsat 8 Operational Land Imager satellite imagery processed with Google Earth Engine cloud‐based geospatial processing platform (Gorelick et al., 2017). This approach estimated the proportion of water contained within individual pixels making it possible to identify small and shallowly flooded emergent wetlands (Halabisky et al., 2016) that we identified as common throughout observed Cinnamon Teal migration pathways. All visible, infrared, and shortwave infrared Landsat bands were included in the SMA, excluding the coastal aerosol band. Surface water estimations represented mean wetland condition assessed from all cloud‐masked Landsat 8 images collected between 1 August and 31 October in the calendar year that a given stopover site was identified. All pixels with >10% water were classified as flooded to capture areas of dense emergent vegetation that can mask underlying flooding commonly used by Cinnamon Teal (DeVries et al., 2017; Thorn & Zwank, 1993). Wetland model identification using this technique, within the same regions we analyzed and during overlapping time periods, has been reported as 93%–98% accurate (Donnelly et al., 2019, 2020). However, we did not conduct an independent accuracy assessment due to delays in transmission of GPS data via the cellular network and an inability to reach remote sites.

**FIGURE 3:**
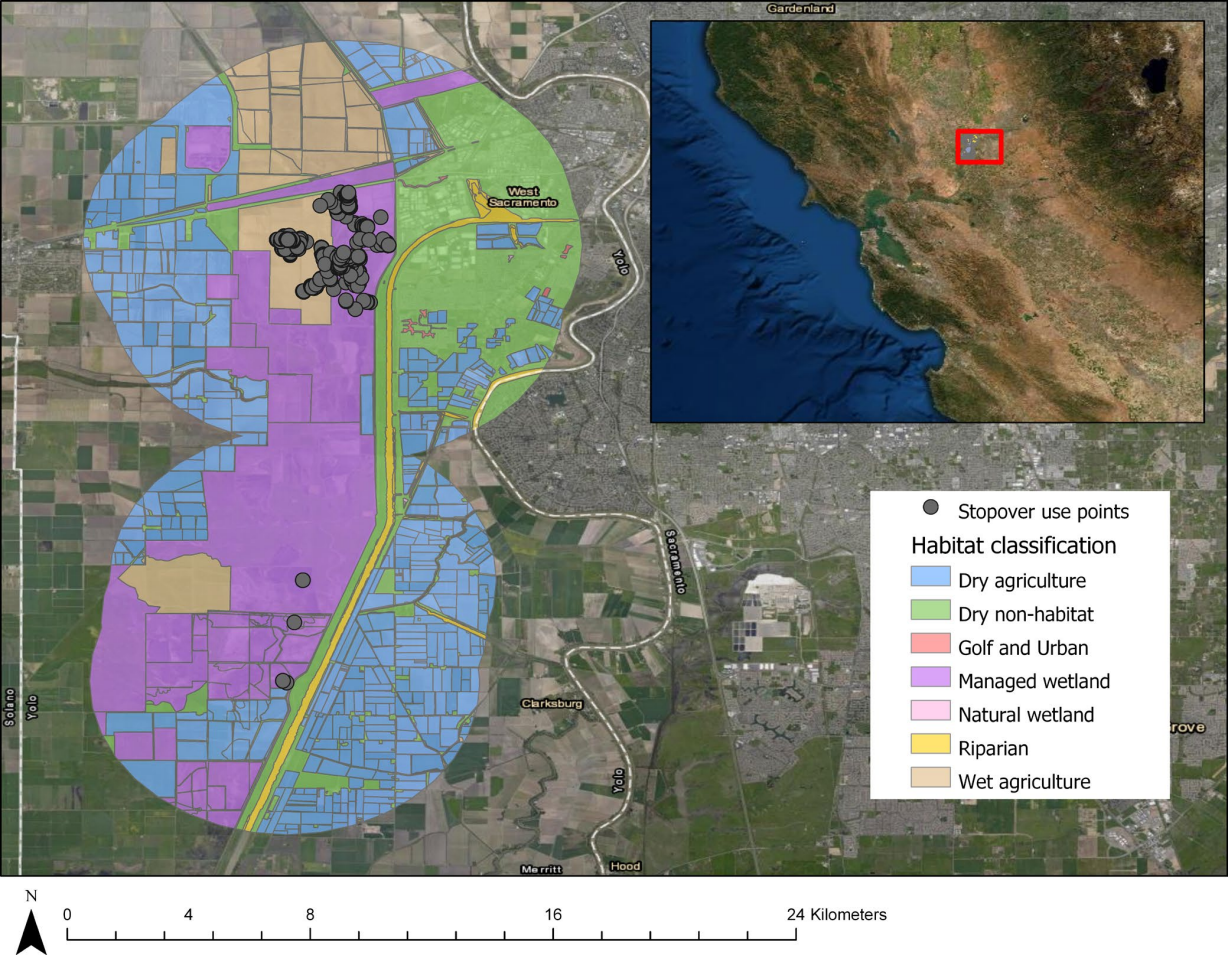
Example of a fall migration stopover site for North American Cinnamon Teal tracked by GPS during the 2017–2019 study. Habitats were delineated within boundary polygons created by buffering stopover use points by 5 km. All areas within the boundaries of those polygons were classified according to the specific habitat type as indicated by legend colors

Wetlands identified by the SMA were summarized using polygons and classified into 10 wetland types: golf and urban; lake and riparian; managed wetland; natural wetland; ocean; riparian; shrimp farm; tidal; wastewater; and wet agriculture (Table 1). Wetland types were identified using photograph interpretation and ancillary GIS data depicting ownership as public or private using the Bureau of Land Management surface ownership layer (BLM, 2021). All areas identified in Mexico were assumed to be private and unprotected (source: personal communication with former Ducks Unlimited Mexico staff biologist, Antonio Cantú). Additionally, we masked known nonflooded agricultural fields in some regions to reduce the extent of area being run in the spectral mixture analysis model. We included these fields as a class (dry agriculture) because we were interested in whether Cinnamon Teal were selecting or avoiding this land cover type. The minimum mapped wetland unit size was 0.25 hectares. All areas within each 5 km stopover buffer were categorized, including regions where no wetlands or agriculture were identified, which we classified as dry nonhabitat (Table 1, Figure 3).

### 2.6 Habitat selection analysis

Third‐order habitat selection within each stopover site (Johnson, 1980) was evaluated using the widesIII model from the R package *adehabitatHS* in R version 4.0.0 (Calenge, 2006; R Core Team, 2020). The model calculates the Manly selectivity measure “*w_i_*” for each input—in our case individual stopover sites—and selection is tested using a chi‐square statistic (Calenge, 2006; Manly et al., 2002). Stopover sites from multiple birds that overlapped were analyzed separately. Use and availability are required inputs for the model and were measured at the stopover level. Area of each land cover type was calculated within the 5 km boundary. Available habitat was defined as the proportion of each land cover type within each stopover site. Using the spatial join tool in ArcGIS Pro™ (version 1.3), we associated each stopover GPS location with its underlying habitat classification. We analyzed each stopover individually, defining “use” as the proportion of stopover locations falling within each habitat type in the stopover site. Cinnamon Teal often use habitat edges (Baldassarre, 2014) that likely increased the potential of slight misalignments between bird GPS locations and wetland habitat identified by the SMA. Although 75% of locations fell within a classified wetland habitat boundary, an additional 19% of locations fell within 100 m (~3 pixels of Landsat derived habitat maps) of classified wetland habitat, indicating that Cinnamon Teal use of wetland edges was high (Appendix S1: Figure A1.2). For these reasons, we identified the nearest classified wetland habitat type for locations falling outside of identified wetlands but occurring within 100 m and attributed this classification to these points.

Selection ratios were first calculated across all stopovers. Percentages of availability and use were reported for each habitat type, along with the Manly selectivity measure (*w_i_*), standard error, and the lower and upper 95% confidence levels (LCL and UCL, respectively) (Mackell et al., 2021). Selection was indicated when LCL was >1 and avoidance indicated when UCL was <1. Lower 95% confidence limit values were truncated at 0.00 because negative values for selection indices are impossible (Kruse et al., 2017).

**FIGURE 4:**
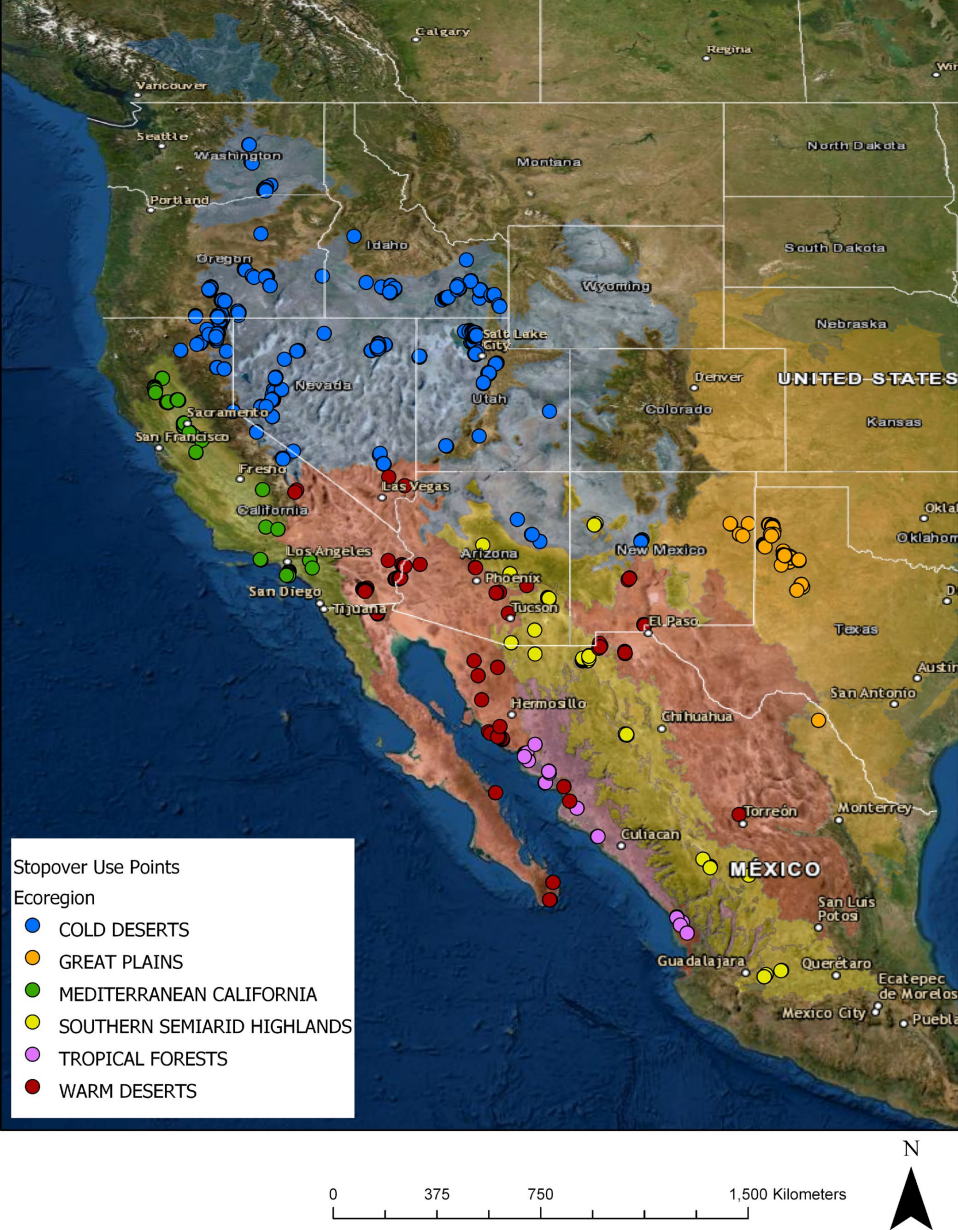
Map showing all non‐flying stopover use points by fall migrating Cinnamon Teal in the years 2017–2019, across all six ecoregions analyzed in our study (Omernik & Griffith, 2014)

To compare regional differences in habitat use, we further defined stopovers by a combination of the level 1 and level 2 ecoregions in which they occurred, as laid out by the Commission for Environmental Cooperation (1999) and defined by Omernik and Griffith (2014), and computed the *w_i_* selection ratios in each region. This provided a useful framework to link physical, biological, and anthropogenic dynamics (e.g., elevation, climate, plant composition, and agricultural development) to the ecology of the landscape. The ecoregions framework was built to serve as a spatial tool for biological assessment and management (Omernik & Griffith, 2014). Ecoregions are not constrained by local, state, or national boundaries, but instead by the environmental and biological properties that make each region distinct. This allowed us to consider the ecological factors that went into shaping the landscape at a coarse spatial scale which was relevant to the bird's ability to make large movements in a short period of time. Regions containing stopover sites included the Cold Deserts (*n* = 96), Great Plains (*n* = 15), Mediterranean California (*n* = 16), Southern Semi‐arid Highlands (*n* = 16), Tropical Forests (*n* = 12), and Warm Deserts (*n* = 31) (Figure 4). In order to accurately represent spatial groupings of waterfowl habitats and improve sample sizes available for analysis, we grouped some ecoregion classifications with adjacent areas where habitat was contiguous. Specifically, stopovers in the Klamath basin of Southern Oregon and Northeastern California are located in the Northwestern Forested Mountains ecoregion but were analyzed with the adjacent Cold Deserts ecoregion. Similarly, two stopovers occurring at the tip of the Baja Peninsula were included with Warm Deserts, and four stopovers occurring in the Temperate Sierras were grouped with the adjacent Southern Semi‐Arid Highlands. Lastly, Tropical Forests was comprised of 10 stopovers from Tropical Dry Forests and two nearby stopovers from Tropical Wet Forests.

## 3 RESULTS

### 3.1 Patterns of migration

Of the 61 individuals that we analyzed, onset of migration was recorded for all except one, whose departure date could not be accurately determined due to a large gap in data (23 days) that coincided with departure (Appendix S2: Table A2.1). For the rest of the birds, the greatest time difference between the last summer location and the first migratory movement location was 14 hr. Across all birds, median departure date was 28 September. Among our observed migration onset dates, we estimated an average departure 1.43 days earlier for each 1 degree increase in latitude but this increase was not significant (SE 1.068, *df* = 57, *p* = .186) due to large individual and annual variation across the range of our data. In addition to median arrival date within each ecoregion, we report the interquartile range and the median absolute deviation to describe variability in these measures (Table 2).

**TABLE 2:** Median arrival dates (day of year) at stopover sites, averaged across each ecoregion. Results include the interquartile range (IQR) and the median absolute deviation (MAD)

Ecoregion	Number of stopovers	Median arrival date (day of year)	IQR	MAD
Cold Deserts	96	267.0	34.75	26.69
Great Plains	15	284.0	20.00	17.79
Mediterranean California	16	286.0	22.00	15.57
Southern Semi‐arid Highlands	16	302.0	32.75	25.95
Tropical Forests	12	303.5	11.75	8.15
Warm Deserts	31	295.0	16.00	11.86

### 3.2 Habitat selection

#### 3.2.1 All stopovers

**FIGURE 5:**
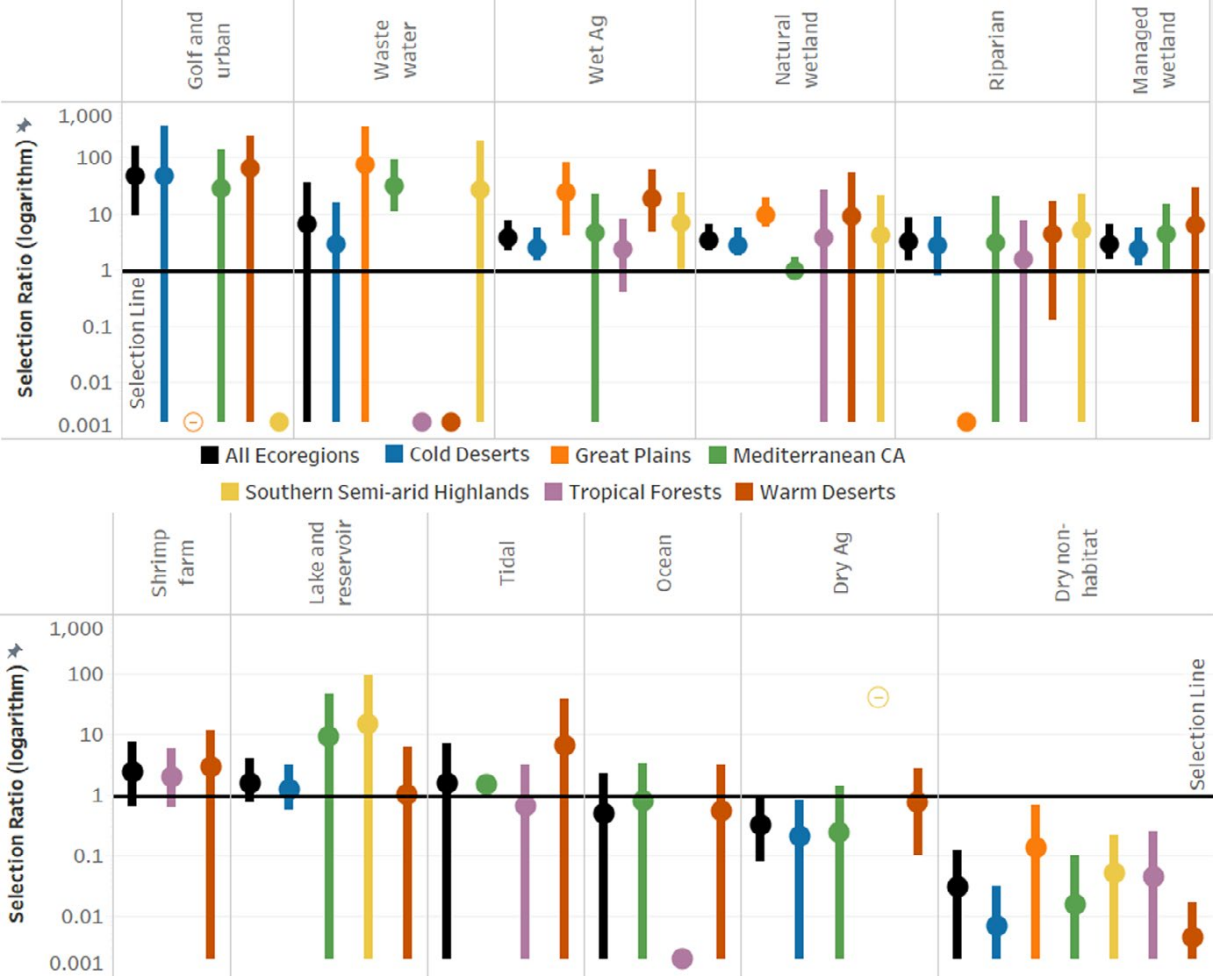
Bars representing the selection ratios and 95% CIs for each ecoregion across all habitat types. Bars that fall above and do not intersect the black horizontal selection ratio line (selection ratio = 1) indicate significant selection for that habitat type. Bars that fall below and do not intersect the selection ratio line indicate significant avoidance of that habitat type. Hollow circles represent instances where only one stopover site indicated selection/avoidance, and therefore, we could not estimate variance for this selection ratio, and we caution interpretation of this reported value. Additionally, solid circles with no bars represent instances where habitat availability was extremely low, there was no use, and selection/avoidance could not be determined

We identified 186 stopover locations from 56 individuals between 2017 and 2019, comprising of 54,478 use points (nonflight), 72.5% of which were on private land. The remaining points were on a mixture of state, federal, and public land. Cinnamon Teal did not select stopover habitat types in proportion to their availability (Figure 5, Appendix S2: Table A2.2). Across all ecoregions, we found selection for (in descending order) golf and urban, (*w_i_* = 46.84), followed by wet agriculture (*w_i_* = 3.66), natural wetland (*w_i_* = 3.36), riparian (*w_i_* = 3.22), and managed wetland (*w_i_* = 2.82). Cinnamon Teal avoided dry nonhabitat (*w_i_* = 0.03) and dry agriculture types (*w_i_* = 0.32). Additionally, dry nonhabitat was avoided in each ecoregion independently.

#### 3.2.2 Cold deserts

The highest number of stopover sites identified was in the Cold Deserts ecoregion (*n* = 96) with a median arrival date of 24 September. Managed wetland, natural wetland, and wet agriculture were all selected for. Of these, natural wetland had the highest selection ratio (*w_i_* = 2.78), followed by wet agriculture (*w_i_* =2.53) and managed wetland (*w_i_* =2.30). There was no ocean, shrimp farm, or tidal area, identified as available in any of the stopover sites. Dry agriculture was avoided (*w_i_* = 0.21).

#### 3.2.3 Great plains

We identified 15 stopovers in the Great Plains ecoregion with a median arrival date of 11 October. Natural wetland (*w_i_* =9.43) and wet agriculture (*w_i_* =23.95) were selected for. There was no dry agriculture, lake, and reservoir, managed wetland, ocean, shrimp farm, or tidal identified as available in any of the stopover sites in this region. Golf and urban was identified in one stopover site (<1% available area of the stopover), but contained no use points and the available area of habitat was too small for selection/avoidance to be determined.

#### 3.2.4 Mediterranean California

Across all 16 stopover sites in the Mediterranean California ecoregion, the median arrival date was 13 October. Wastewater (*w_i_* = 31.53) and tidal marsh (*w_i_* = 1.44) habitat types were selected. Dry agriculture (*w_i_* = 0.24) was avoided. Shrimp farm was the only habitat type that was not identified as available in any stopover site.

#### 3.2.5 Southern semi‐arid highlands

Sixteen stopover sites were used within the Southern Semi‐arid Highlands ecoregion with a median arrival date of 29 October. Our selection ratio for dry agriculture in the Semi‐arid Highland ecoregion (Figure 5, Appendix S2: Table A2.1) was represented by a single stopover site with disproportionately high use (31.7% of locations) relative to availability (<1% of stopover habitat). Because we had no additional selection for dry agriculture in this ecoregion, we could not estimate variance for this selection ratio, and we caution interpretation of this reported value. Managed wetland, ocean, shrimp farm, and tidal were not identified as available at any of the stopover sites in this region.

#### 3.2.6 Tropical forests

The stopover sites in the Tropical Forests ecoregion (*n* = 12) had a median arrival date of 30 October. We detected no significant selection for any habitat type in this ecoregion. Dry agriculture, golf and urban, lake and reservoir, and managed wetland were not identified as available at any stopover site.

#### 3.2.7 Warm Deserts

The Warm Deserts ecoregion contained 31 stopover sites and had a median arrival date of 22 October. All 12 land cover types were identified in this region but wet agriculture was the only habitat selected for (*w_i_* = 18.46).

## 4 DISCUSSION

Our analysis of fall‐migrating Cinnamon Teal tracked with GPS‐GSM transmitters highlighted selection for wet agriculture, natural wetland, managed wetland, riparian, tidal, wastewater, and golf and urban habitat types at levels disproportionate to the availability of those habitats within the western landscape. By contrast, dry nonhabitat was consistently avoided across all ecoregions and dry agriculture was avoided in the Cold Deserts and Mediterranean California ecoregions. Over 72% of stopovers occurred on private land, and Cinnamon Teal often utilized some of the secondary features associated with agriculture and urbanization (e.g., irrigation ditches, stock ponds, and golf course water features). Moreover, 29.8% of stopover use points occurred in wet agriculture, the most used habitat type across all ecoregions in our study. These findings raise important implications for managing resources for migrating waterfowl and highlight the importance of cooperating with private stakeholders.

We detected significant selection for seven of the 10 wetland habitat types that we classified in our selection analysis. Cinnamon Teal demonstrated a wide range of habitat preferences across landscapes where wetland availability was often limited and highly variable across stopover locations. Despite this variation, patterns of habitat use (both for selection and avoidance) were always in concurrence among ecoregions where patterns were statistically significant. Robinson and Warnock (1997) proposed that because water regimes in areas like the Great Basin are highly variable, migratory birds in this region may be more opportunistic and less committed to local sites. Our results support this assessment, as habitat availability was highly variable across stopovers, resulting in selection for a wide variety of wetland types as birds took advantage of what limited habitat was available. We interpret this in contrast to a “generalist” selection pattern where all habitats are available with little to no selection evident among them. Variation in patterns of observed habitat use is likely driven by the differences in available habitat along migratory routes. Future research analyzing habitat selection at a scale beyond stopover sites (e.g., migratory paths) could provide important insight into potentially opportunistic behavior exhibited by fall‐migrating Cinnamon Teal.

We found that relatively scarce habitat was highly selected, which signifies the importance of these limited resources. For instance, in the Mediterranean California ecoregion, wastewater and tidal marshes, comprising of only 0.4% and 0.73% of available habitat across the stopovers in this ecoregion respectively, were selected for by migratory Cinnamon Teal. Similarly, golf and urban area constituted only 0.2% of available land cover within Warm Desert stopover sites but was also selected for. Golf courses, which have been linked to environmental issues such as the destruction of native landscapes, consuming scarce water resources, and excessive use of pesticides and fertilizers (Pearce, 1993; Wurl, 2019), have also been shown to enhance landscape connectivity and act as wildlife refuges, particularly in urban and agricultural landscapes (Hodgkison et al., 2007; Petrosillo et al., 2019). Our data suggest that golf courses and wastewater ponds can serve as stopover resources for migrating Cinnamon Teal in often dry, anthropogenically altered landscapes, providing opportunities for birds to stop, rest, and refuel.

Our results indicate high selectivity by migrating Cinnamon Teal for habitats which generally have low prevalence across western North America. Donnelly et al. (2019) found abundance of seasonal wetland was most limited during the peak of Cinnamon Teal migration (September through mid‐October). That the lowest values of wetland abundance coincides with peak migration suggests a particular vulnerability of this species to reduced habitat availability, quality, or perturbations in wetland networks that support this life‐history stage. Low abundance of flooded wetlands also raises concerns over future vulnerability to climate change and intensified drought conditions (Haig et al., 2019; Padrón et al., 2020; Williams, Cook, et al., 2020) as well as anthropogenic landscape change (Xu et al., 2021).

Much of waterfowl research has focused on the breeding and wintering grounds but there is a growing understanding among ecologists that habitat availability during migration is essential in linking these two life‐history stages (Bonter et al., 2009; Davis et al., 2014; Williams et al., 2020). Knowing the timing and type of habitats used during migration stopovers helps inform managers when specific habitats in each ecoregion would be required. This is particularly important in western North America which has had, and continues to experience, substantial changes in wetland availability across a landscape that often pits habitat managers, farmers, and urban settlements in competition for increasingly limited water resources (Dettinger et al., 2015; King et al., 2021).

Given the large percentage of privately owned land used by migrating Cinnamon Teal, working with private landowners to meet conservation goals may be a critically important strategy. Programs aimed at cooperating with private landowners have made contributions to bird conservation in North America by working with stakeholders in the farming and ranching community. For example, the Farm Bill allowed the implementation of conservation programs on tens of millions of hectares of agricultural lands, including the restoration of over 930,000 ha of wetlands (Ciuzio et al., 2013). Given the high use of wet agricultural habitats, conservation strategies such as incentivizing farmers to ensure that beneficial agricultural practices (e.g., flooding) align with migration chronology could be a useful and cost‐effective strategy to enhance wetlands and provide the resources necessary for these birds to complete migration. However, this strategy has limitations as fall migration, unlike the spring, does not align with traditional flood irrigation practices across much of the west. Prioritizing fall flooding on public refuges may offset constraints on private land when crop irrigation is minimal. Also, because our results show that Cinnamon Teal also use smaller habitat areas dispersed on the landscape, the potential benefits of implementing conservation on smaller scales could be considered to increase availability of aquatic resources during this vulnerable time.

We highlight the varying importance of different habitats among regions used by migrating Cinnamon Teal. A next step in this research could be to evaluate the quality of used habitats. The observed patterns of habitat selection by Cinnamon Teal do not necessarily imply good habitat quality. Increased predation risk, pollution, and low food quality are important considerations when assessing habitat values. For example, an agriculture ditch may provide valuable feeding opportunities but is potentially a focal point for predators, increasing the potential for depredation. An evaluation of habitat quality, not just use, would enable us to identify further potential threats that these birds may face during migration and assess the potential impacts of reduced habitat quality and availability on populations and movement.

Our selection analysis relied on several assumptions that could not be directly validated empirically. Because we use each stopover site as the sampling unit, we assume selection patterns within each stopover are independent. Individual bias in preferred habitat would be a violation of that assumption that could affect the applicability of our results. However, because habitat availability is independent between stopover sites for a given individual (though multiple individuals could each have overlapping stopover spots), selection ratios should be independent as well. Due to the limited number of individuals with sufficient replicate stopover sites identified, particularly within individual ecoregions, there was not sufficient information to accurately estimate random effect terms for individuals. In addition, we assumed that impacts from transmitter marking did not affect migration chronology and selection patterns for individuals. Although transmitter attachment can affect migrating bird's survival rates (Lameris et al., 2018), energy reserves (Hupp et al., 2015; Pennycuick et al., 2012), and migration chronology (Hupp et al., 2015), negative impacts of migration independent of carrying transmitters are relatively common in numerous bird taxa (Klaassen et al., 2014; Szostek & Becker, 2015; Tavares et al., 2020). We had some individuals for which we never received location data, or they “disappeared” mid‐track. It is possible these individuals died as a result of transmitter attachment, but, given other individuals carried transmitters successfully for lengthy durations and others reappeared after lengthy data gaps, we cannot determine if lack of data is indicative of mortalities, poor connectivity to the cellular networks or transmitter hardware failure.

Habitat provided by effective management has been important in the protection of waterfowl in North America (Anderson et al., 2018). In a recent study of North American avifauna population trends, the Anatidae were one of the few taxa whose numbers had increased across the duration of the study (56% across all species, 1970–2018) (Rosenberg et al., 2019). That success can largely be attributed to active management of migratory species through the Migratory Bird Treaty Act and management of wetlands through the North American Waterfowl Management Plan and the North America Wetland Conservation Act (Anderson et al., 2018; U.S. Department of the Interior et al., 2012). These initiatives specifically target the protection of migratory species and the protection and enhancement of wetland ecosystems on which waterfowl depend. Nonetheless, not all waterfowl species have exhibited positive population trends, and though information on Cinnamon Teal is limited, a recent study identified a population trend from 1966–2015 of −2.074% per year (Sauer et al., 2017). This is especially concerning, given that they are one of the least abundant dabbling ducks in North America (Baldassarre, 2014; Williams et al., 1999). Moreover, the grouping of Cinnamon Teal with Blue‐winged Teal in population surveys complicates accurate population estimates and heightens the importance of our study. Our findings indicate that wetland management and waterfowl migration could be better synchronized and that this effort may depend on developing a diverse array of conservation and management tools through broadscale collaborations and incentive‐based management practices. Such efforts would help preserve often unprotected or overlooked resources that may be critical for the success of this species.

## CONFLICT OF INTEREST

The authors declare no conflicts of interest. This study was approved by the U.S. Geological Survey Western Ecological Research Center Animal Care and Use Committee and conducted under Federal Banding Permit #21142 and state SC permit #SC‐8090.

## AUTHOR CONTRIBUTION

**Desmond A. Mackell:** Conceptualization (equal); Data curation (equal); Formal analysis (equal); Investigation (equal); Methodology (equal); Visualization (equal); Writing‐original draft (lead); Writing‐review & editing (equal). **Michael L. Casazza:** Conceptualization (equal); Data curation (equal); Formal analysis (supporting); Funding acquisition (lead); Investigation (equal); Methodology (supporting); Project administration (equal); Resources (lead); Writing‐original draft (supporting); Writing‐review & editing (equal). **Cory T. Overton:** Data curation (equal); Formal analysis (equal); Investigation (equal); Methodology (equal); Visualization (equal); Writing‐original draft (supporting); Writing‐review & editing (equal). **J. Patrick Donnelly:** Formal analysis (equal); Investigation (equal); Methodology (equal); Visualization (equal); Writing‐review & editing (equal). **David Olson:** Conceptualization (equal); Funding acquisition (lead); Investigation (equal); Writing‐review & editing (equal). **Fiona McDuie:** Writing‐original draft (supporting); Writing‐review & editing (equal). **Joshua T. Ackerman:** Formal analysis (supporting); Funding acquisition (supporting); Resources (supporting); Writing‐review & editing (equal). **John M. Eadie:** Conceptualization (equal); Funding acquisition (supporting); Writing‐original draft (supporting); Writing‐review & editing (equal).

## Supporting information


**Figure A1.1.** The number of GPS locations collected from 61 individual fall migrating Cinnamon Teal at sampling rates that varied from 15 minutes to 6 hours. Study was conducted across the western United States and Mexico (see Fig. 1) during the years 2017‐19.
**Figure A1.2.** Percentage of GPS locations that would be included in classified wetlands with various distances to wetlands. As distance to wetlands increases the number of included locations asymptotes. A 100m boundary around wetlands included 94% of all stopover use locations so this was selected as the buffer distance to account for any error in wetland identification and GPS error of the transmitters, and each point within that distance (red dashed line) of a classified wetland was assigned to its nearest classified wetland type.

## Data Availability

The datasets generated during the current study are available in the ScienceBase repository (https://doi.org/10.5066/P99L4XJ5).
