# Supplementary material for: Migration stopover ecology of Cinnamon Teal in western North America
Source: Ecol Evol. 2021 Sep 21;11(20):14056–69. doi: 10.1002/ece3.8115 (PMC8525093; doi:10.1002/ece3.8115)
Supplement: Supplementary file 1 — Figure A1.1. The number of GPS locations collected from 61 individual fall migrating Cinnamon Teal at sampling rates that varied from 15 minutes to 6 hours. Study was conducted across the western United States and Mexico (see Fig. 1) during the years 2017‐19. Figure A1.2. Percentage of GPS locations that would be included in classified wetlands with various distances to wetlands. As distance to wetlands increases the number of included locations asymptotes. A 100m boundary around wetlands included 94% of all stopover use locations so this was selected as the buffer distance to account for any error in wetland identification and GPS error of the transmitters, and each point within that distance (red dashed line) of a classified wetland was assigned to its nearest classified wetland type. [file ECE3-11-14056-s001.docx]

**Appendix S1:** **Figures**


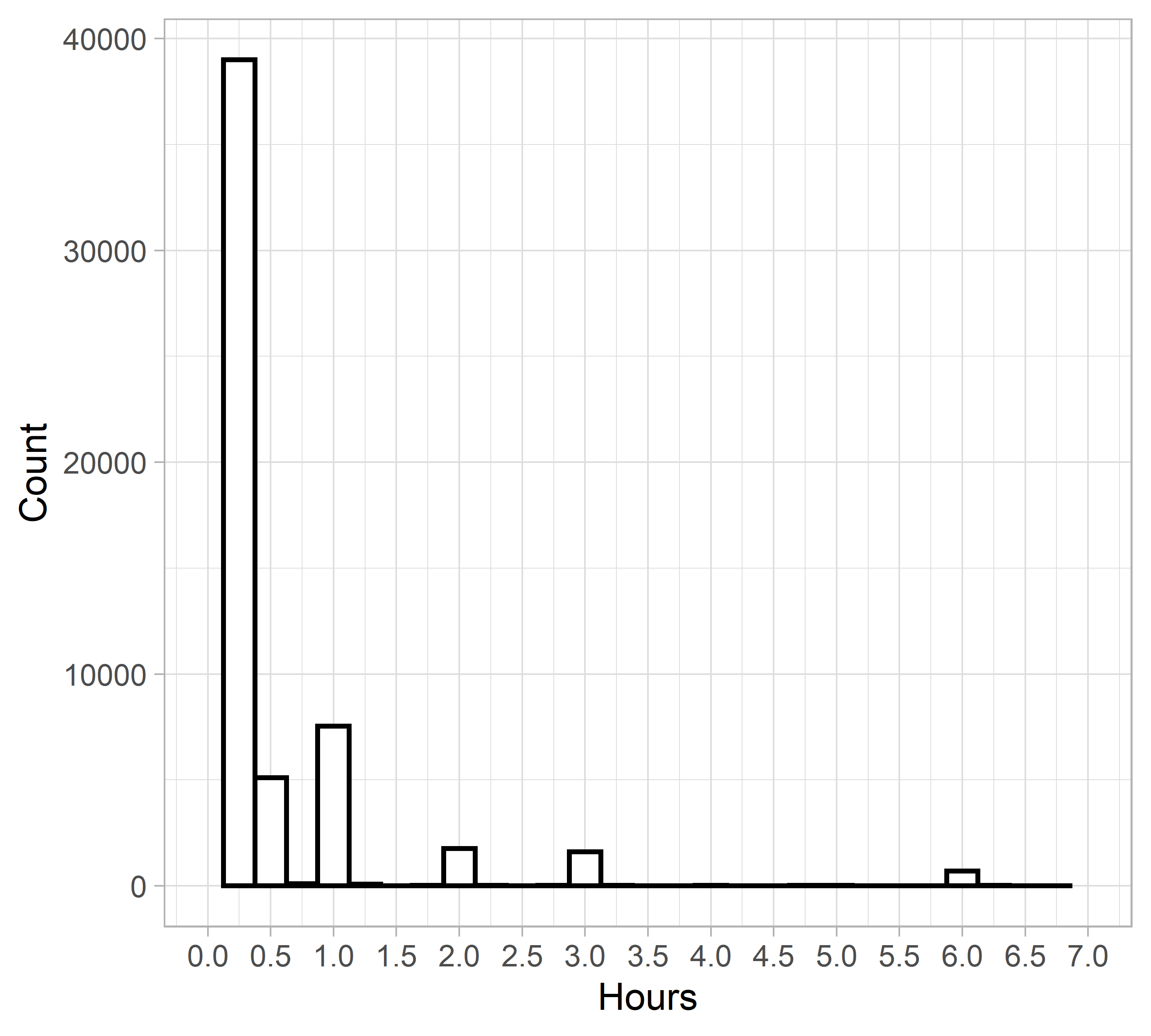


Figure A1.1. The number of GPS locations collected from 61 individual fall migrating Cinnamon Teal at sampling rates that varied from 15 minutes to 6 hours. Study was conducted across the western United States and Mexico (see Fig. 1) during the years 2017-19.

| 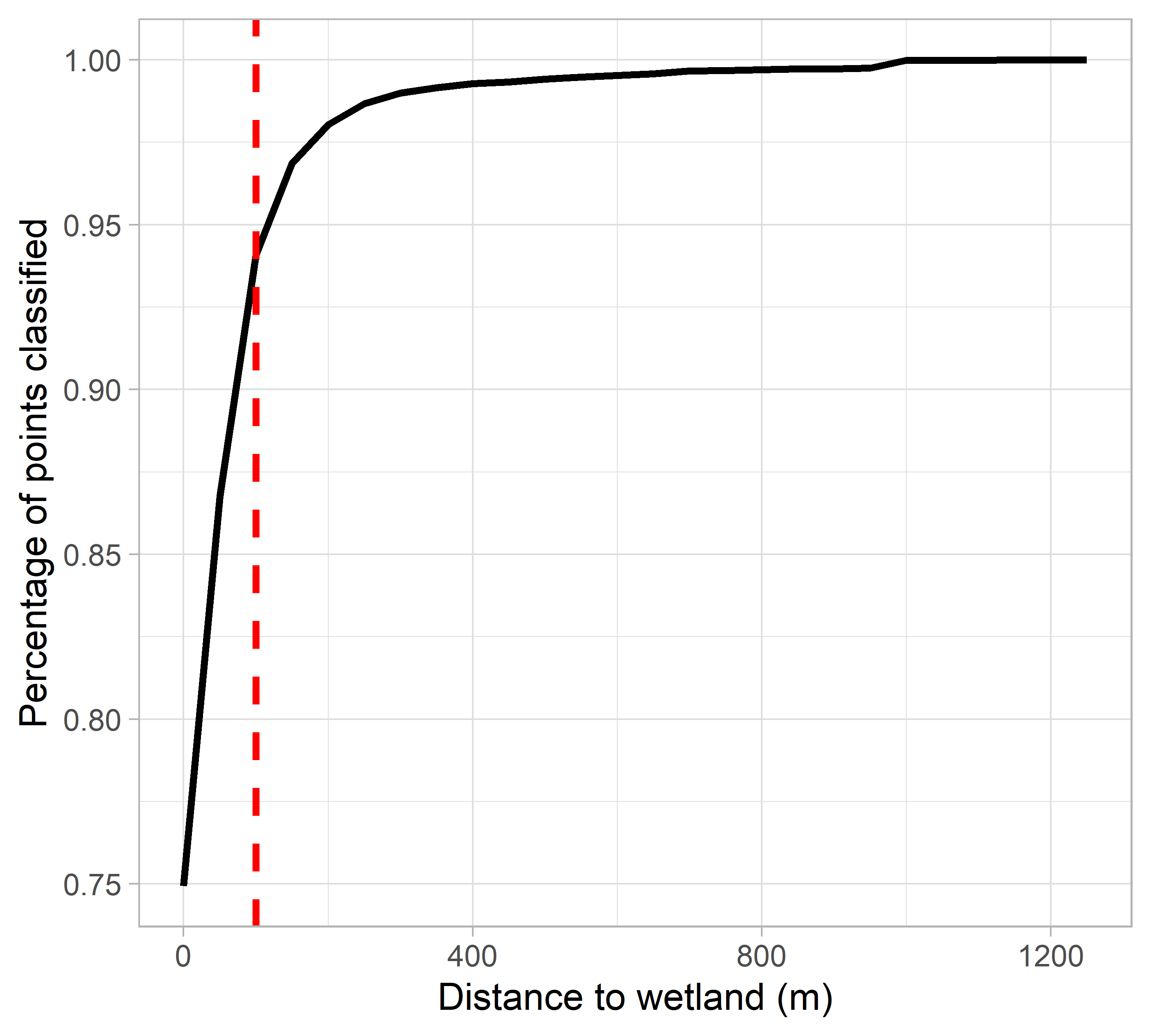 |  |  |  |  |
| --- | --- | --- | --- | --- |

Figure A1.2. Percentage of GPS locations that would be included in classified wetlands with various distances to wetlands. As distance to wetlands increases the number of included locations asymptotes. A 100m boundary around wetlands included 94% of all stopover use locations so this was selected as the buffer distance to account for any error in wetland identification and GPS error of the transmitters, and each point within that distance (red dashed line) of a classified wetland was assigned to its nearest classified wetland type.

**Appendix S2:** **Tables**

Table A2.1 Marking and route information for all migrating Cinnamon Teal. AHY - After Hatch Year, HY – Hatch Year. Migration Distance is presented as the net displacement from marking location to the end of the bird’s track. For individuals that did not complete migration, the last known live location was used and the end of track date.

| **Bird ID** | **Age** | **Sex** | **Marking Location** | **Year** | **Migration Start** | **End of Track** | **# of Stopovers** | **Average Duration (d)** | **Migration Distance (km)** | **Completed Migration** |
| --- | --- | --- | --- | --- | --- | --- | --- | --- | --- | --- |
| 191529.1 | AHY | F | Klamath | 2019 | 9-Oct | 19-Oct | 4 | 2.6 | 306.8 | Unknown |
| 191534.1 | AHY | F | Klamath | 2019 | 29-Sep | 28-Oct | 2 | 14.1 | 779.7 | Yes |
| 191537.1 | AHY | F | Suisun | 2019 | 5-Oct | 6-Dec | 6 | 10.0 | 2970.3 | Yes |
| 191538.1 | AHY | F | Klamath | 2019 | 17-Oct | 28-Oct | 2 | 5.3 | 415.3 | Unknown |
| 191551.1 | AHY | M | Summer Lake | 2019 | 31-Aug | 8-Oct | 4 | 9.6 | 436.7 | Unknown |
| 191552.1 | AHY | M | Summer Lake | 2019 | 4-Oct | 5-Oct | 1 | 0.7 | 767.6 | Unknown |
| 191553.1 | AHY | M | Summer Lake | 2019 | 5-Sep | 5-Oct | 5 | 5.9 | 734.4 | No |
| 191556.1 | AHY | F | Summer Lake | 2019 | 13-Oct | 15-Oct | 2 | 0.6 | 775.3 | Yes |
| 191558.1 | AHY | M | Summer Lake | 2019 | 26-Aug | 27-Sep | 2 | 13.9 | 437.3 | Unknown |
| 191559.1 | AHY | M | Summer Lake | 2019 | 20-Sep | 28-Oct | 3 | 12.5 | 580.7 | Unknown |
| 191562.1 | HY | M | Mud Lake | 2019 | 12-Sep | 19-Sep | 1 | 7.2 | 390.1 | No |
| 191564.1 | HY | F | Mud Lake | 2019 | 2-Oct | 24-Oct | 3 | 7.3 | 698.5 | No |
| 191567.1 | AHY | M | Salt Lake | 2019 | 30-Oct | 2-Nov | 1 | 1.7 | 565.5 | No |
| 191575.1 | AHY | M | Salt Lake | 2019 | 24-Oct | 26-Oct | 1 | 0.8 | 1757.5 | Yes |
| 191578.1 | AHY | F | Klamath | 2019 | 27-Oct | 27-Oct | 0 |  | 531.4 | Yes |
| 191582.1 | AHY | F | Sac Valley | 2019 | 19-Sep | 23-Oct | 4 | 8.3 | 762.3 | Unknown |
| 191583.1 | AHY | F | Sac Valley | 2019 | 24-Oct | 25-Dec | 5 | 12.2 | 1015.4 | Yes |
| 191588.1 | HY | M | Trueblood | 2019 | 19-Sep | 24-Sep | 2 | 2.2 | 290.6 | No |
| 191589.1 | HY | M | Trueblood | 2019 | 18-Oct | 19-Oct | 1 | 0.5 | 1316 | Yes |
| 191590.1 | AHY | F | Trueblood | 2019 | 18-Aug | 10-Nov | 6 | 13.9 | 1696.3 | Unknown |
| 191615.1 | AHY | F | Swan Lake | 2019 | 18-Sep | 16-Oct | 1 | 27.9 | 624.1 | No |
| 191624.1 | AHY | M | Stillwater | 2019 | Unknown | 8-Nov | 4 | 8.9 | 2018.5 | Unknown |
| 191625.1 | AHY | M | Stillwater | 2019 | 20-Sep | 30-Sep | 2 | 4.7 | 1337.9 | No |
| DRIB 49.1 | AHY | F | Suisun | 2017 | 18-Nov | 18-Nov | 0 |  | 440.5 | Yes |
| FOWL 02.1 | AHY | M | Salt Lake | 2017 | 26-Sep | 11-Oct | 3 | 4.6 | 1171.3 | No |
| FOWL 07.1 | AHY | M | Salt Lake | 2017 | 27-Sep | 28-Oct | 4 | 8.9 | 2240.5 | Yes |
| FOWL 10.1 | AHY | F | Salt Lake | 2017 | 9-Oct | 11-Oct | 0 |  | 1710.6 | No |
| FOWL 12.1 | AHY | F | Coeur d'Alene | 2017 | 1-Oct | 15-Oct | 4 | 3.3 | 1975.4 | Unknown |
| FOWL 13.1 | AHY | F | Coeur d'Alene | 2017 | 24-Oct | 7-Nov | 2 | 6.7 | 546.8 | No |
| FOWL 15.1 | HY | M | Coeur d'Alene | 2017 | 1-Oct | 30-Oct | 4 | 6.7 | 1925 | Unknown |
| FOWL 16.1 | HY | M | Coeur d'Alene | 2017 | 4-Nov | 7-Nov | 1 | 0.5 | 1576.8 | Yes |
| FOWL 17.1 | HY | F | Coeur d'Alene | 2017 | 23-Oct | 28-Oct | 2 | 1.8 | 1490.4 | Unknown |
| SIMP 12.1 | AHY | F | Coeur d'Alene | 2018 | 14-Oct | 11-Nov | 4 | 4.0 | 1913.4 | No |
| SIMP 23.1 | AHY | F | Mud Lake | 2018 | 24-Sep | 4-Nov | 3 | 13.2 | 1282.3 | No |
| SIMP 28.1 | AHY | M | Mud Lake | 2018 | 28-Aug | 17-Nov | 15 | 4.8 | 2540.9 | Unknown |
| SIMP 29.1 | AHY | M | Mud Lake | 2018 | 30-Aug | 10-Nov | 2 | 9.4 | 848.7 | No |
| SIMP 31.1 | AHY | M | Summer Lake | 2018 | 28-Sep | 26-Nov | 4 | 14.0 | 1194.3 | Yes |
| SIMP 33.1 | AHY | F | Mud Lake | 2018 | 8-Oct | 10-Oct | 2 | 1.0 | 191 | No |
| SIMP 37.1 | AHY | M | Summer Lake | 2018 | 4-Sep | 19-Dec | 7 | 14.6 | 2695.9 | Yes |
| SIMP 39.1 | AHY | M | Summer Lake | 2018 | 25-Sep | 4-Nov | 1 | 38.8 | 228.9 | No |
| SIMP 41.1 | AHY | M | Summer Lake | 2018 | 23-Sep | 9-Oct | 3 | 4.8 | 1049.8 | No |
| SIMP 42.1 | AHY | F | Summer Lake | 2018 | 7-Sep | 23-Oct | 5 | 8.9 | 733.4 | Unknown |
| SIMP 44.1 | AHY | F | Summer Lake | 2018 | 3-Sep | 6-Nov | 4 | 15.9 | 649 | Unknown |
| SIMP 55.1 | AHY | M | Monte Vista | 2018 | 11-Oct | 17-Dec | 6 | 9.0 | 737.2 | Yes |
| SIMP 62.1 | AHY | F | Sac Valley | 2019 | 2-Sep | 23-Oct | 5 | 4.8 | 1671.8 | No |
| TEAL 03.1 | AHY | M | Stillwater | 2017 | 28-Nov | 28-Nov | 0 |  | 599.5 | Yes |
| TEAL 07.1 | HY | M | Worth Lake | 2017 | 14-Sep | 15-Sep | 1 | 0.7 | 554.4 | No |
| TEAL 09.1 | AHY | F | Worth Lake | 2017 | 4-Nov | 8-Nov | 3 | 1.0 | 654.4 | No |
| TEAL 24.2 | AHY | F | Suisun | 2018 | 13-Aug | 26-Nov | 6 | 17.3 | 1513 | Yes |
| TEAL 34.1 | AHY | M | Summer Lake | 2017 | 31-Oct | 2-Nov | 1 | 0.7 | 1250.2 | Yes |
| TEAL 35.1 | AHY | M | Summer Lake | 2017 | 8-Sep | 17-Sep | 2 | 4.6 | 501.8 | No |
| TEAL 37.1 | AHY | M | Summer Lake | 2017 | 9-Sep | 10-Sep | 0 |  | 386.3 | Yes |
| TEAL 38.1 | AHY | M | Summer Lake | 2017 | 19-Aug | 20-Sep | 2 | 15.3 | 613.7 | Unknown |
| TEAL 39.1 | AHY | F | Summer Lake | 2017 | 3-Sep | 14-Oct | 5 | 8.1 | 798.7 | Unknown |
| TEAL 39.2 | AHY | M | Salt Lake | 2018 | 18-Nov | 20-Nov | 2 | 0.5 | 773.7 | Yes |
| TEAL 40.1 | AHY | F | Summer Lake | 2017 | 12-Oct | 15-Oct | 2 | 0.9 | 1145.1 | Unknown |
| TEAL 50.1 | AHY | M | Salt Lake | 2018 | 8-Nov | 15-Nov | 3 | 1.8 | 1194.2 | Unknown |
| TEAL 54.2 | AHY | M | Salt Lake | 2018 | 7-Oct | 2-Nov | 5 | 4.8 | 1723.3 | Unknown |
| TEAL 61.1 | AHY | M | Monte Vista | 2017 | 7-Oct | 9-Oct | 1 | 0.7 | 1364.6 | Yes |
| TEAL 63.1 | AHY | M | Monte Vista | 2017 | 30-Aug | 31-Aug | 1 | 0.7 | 472.7 | No |
| TEAL 66.1 | AHY | M | Monte Vista | 2017 | 28-Aug | 28-Oct | 9 | 7.6 | 1149 | No |

Table A2.2. Results for habitat usage for 56 individual Cinnamon Teal studied across the western United States and Mexico. Of the 186 individual stopover sites identified in this study spanning the years 2017-19, we present the number of stopover sites associated with the each of the six North American ecoregions they fell in as defined by Omernik and Griffith (2014). Within each ecoregion, we present the proportion (%) of habitat available, % used, and define selection (w_i_ ±SE and 95% LCL and UCL) for 12 classified habitat types (see Table 2 for descriptions). ***** indicates selection (95% CI of w_i_ >1), ** indicates avoidance (95% CI of w_i_ <1). † Negative values were replaced with 0.00 because negative values for selection indices are impossible. Results are presented for all North American ecoregions grouped together and for each ecoregion separately.

| **Ecoregions** | **Habitat type** | **Dry agriculture** | **Dry non-habitat** | **Golf and urban** | **Lake and reservoir** | **Managed wetland** | **Natural wetland** | **Ocean** | **Riparian** | **Shrimp farm** | **Tidal** | **Wastewater** | **Wet agriculture** |
| --- | --- | --- | --- | --- | --- | --- | --- | --- | --- | --- | --- | --- | --- |
| **All ecoregion stopovers (n=186)** | **% Available** | 9.45 | 57.65 | 0.07 | 6.47 | 4.39 | 7.75 | 2.20 | 2.11 | 0.38 | 0.91 | 0.49 | 8.11 |
|  | **% Used** | 2.99 | 1.77 | 3.21 | 10.4 | 12.39 | 26.06 | 1.08 | 6.80 | 9.41 | 1.46 | 3.19 | 29.70 |
|  | **w_i_** | 0.32** | 0.03** | 46.84* | 1.61 | 2.82* | 3.36* | 0.49 | 3.22* | 2.45 | 1.59 | 6.46 | 3.66* |
|  | **SE** | 0.08 | 0.01 | 13.10 | 0.29 | 0.44 | 0.39 | 0.24 | 0.60 | 0.62 | 0.76 | 3.95 | 0.49 |
|  | **95% LCL** | 0.08 | 0.00† | 9.31 | 0.78 | 1.57 | 2.25 | 0.00† | 1.50 | 0.68 | 0.00† | 0.00† | 2.26 |
|  | **95% UCL** | 0.55 | 0.06 | 84.36 | 2.44 | 4.07 | 4.47 | 1.18 | 4.93 | 4.22 | 3.76 | 17.78 | 5.06 |
| **Cold Deserts (n=96)** | **% Available** | 9.92 | 47.22 | < 0.01 | 11.02 | 6.89 | 12.89 | 0 | 1.89 | 0 | 0 | 0.69 | 9.44 |
|  | **% Used** | 2.06 | 0.33 | 1.04 | 13.70 | 15.84 | 35.87 | 0 | 5.25 | 0 | 0 | 2.05 | 23.86 |
|  | **w_i_** | 0.21** | 0.006** | 46.07 | 1.24 | 2.30* | 2.78* | NA | 2.78 | NA | NA | 2.95 | 2.53* |
|  | **SE** | 0.08 | 0.003 | 49.22 | 0.24 | 0.40 | 0.34 | NA | 0.72 | NA | NA | 1.82 | 0.39 |
|  | **95% LCL** | 0.00† | 0.00† | 0.00† | 0.59 | 1.20 | 1.84 | NA | 0.78 | NA | NA | 0.00† | 1.44 |
|  | **95% UCL** | 0.42 | 0.02 | 182.54 | 1.90 | 3.40 | 3.73 | NA | 4.78 | NA | NA | 8.00 | 3.61 |
| **Great Plains (n=16)** | **% Available** | 0 | 91.93 | < 0.01 | 0 | 0 | 6.31 | 0 | 0.84 | 0 | 0 | 0.12 | 0.78 |
|  | **% Used** | 0 | 12.17 | 0 | 0 | 0 | 59.56 | 0 | 0 | 0 | 0 | 9.57 | 18.70 |
|  | **w_i_** | NA | 0.13** | 0 | NA | NA | 9.43* | NA | 0 | NA | NA | 77.00 | 23.95* |
|  | **SE** | NA | 0.08 | 0 | NA | NA | 1.27 | NA | 0 | NA | NA | 37.07 | 7.47 |
|  | **95% LCL** | NA | 0.00† | 0 | NA | NA | 6.08 | NA | 0 | NA | NA | 0.00† | 4.24 |
|  | **95% UCL** | NA | 0.34 | 0 | NA | NA | 12.79 | NA | 0 | NA | NA | 174.79 | 43.66 |
| **Mediterranean California (n=16)** | **% Available** | 34.46 | 42.13 | 0.23 | 1.40 | 6.57 | 0.90 | 7.68 | 2.35 | 0 | 0.73 | 0.40 | 3.15 |
|  | **% Used** | 8.17 | 0.67 | 6.51 | 12.90 | 29.41 | 0.85 | 6.25 | 7.01 | 0 | 1.08 | 12.48 | 14.67 |
|  | **w_i_** | 0.24** | 0.02** | 28.00 | 9.22 | 4.47 | 0.95 | 0.81 | 2.99 | NA | 1.49* | 31.53* | 4.65 |
|  | **SE** | 0.17 | 0.01 | 15.38 | 5.17 | 1.25 | 0.09 | 0.30 | 2.49 | NA | 0.004 | 7.17 | 2.43 |
|  | **95% LCL** | 0.00† | 0.00† | 0.00† | 0.00† | 0.92 | 0.69 | 0.00† | 0.00† | NA | 1.48 | 11.19 | 0.00† |
|  | **95% UCL** | 0.73 | 0.05 | 71.63 | 23.89 | 8.02 | 1.22 | 1.67 | 10.06 | NA | 1.50 | 51.86 | 11.56 |
| **Southern Semi-arid Highlands (n=16)** | **% Available** | 0.05 | 86.25 | 0.03 | 1.24 | 0 | 2.88 | 0 | 2.94 | 0 | 0 | 0.13 | 6.49 |
|  | **% Used** | 1.98 | 4.51 | 0 | 19.12 | 0 | 11.91 | 0 | 14.75 | 0 | 0 | 3.36 | 44.36 |
|  | **w_i_** | 41.39* | 0.05** | 0 | 15.43 | NA | 4.14 | NA | 5.01 | NA | NA | 26.57 | 6.84 |
|  | **SE** | 0 | 0.02 | 0 | 12.50 | NA | 2.54 | NA | 2.37 | NA | NA | 26.56 | 2.18 |
|  | **95% LCL** | 41.39 | 0.00† | 0 | 0.00† | NA | 0.00† | NA | 0.00† | NA | NA | 0.00† | 0.88 |
|  | **95% UCL** | 41.39 | 0.11 | 0 | 49.60 | NA | 11.07 | NA | 11.49 | NA | NA | 98.93 | 12.79 |
| **Tropical Forests (n=12)** | **% Available** | 0 | 41.48 | 0 | 0 | 0 | 2.12 | 8.30 | 2.45 | 3.08 | 11.25 | 0.08 | 31.25 |
|  | **% Used** | 0 | 1.86 | 0 | 0 | 0 | 7.97 | 0 | 3.89 | 6.25 | 7.57 | 0 | 72.46 |
|  | **w_i_** | NA | 0.04** | NA | NA | NA | 3.76 | 0 | 1.59 | 2.03 | 0.67 | 0 | 2.32 |
|  | **SE** | NA | 0.03 | NA | NA | NA | 3.43 | 0 | 0.85 | 0.51 | 0.36 | 0 | 0.70 |
|  | **95% LCL** | NA | 0.00† | NA | NA | NA | 0.00† | 0 | 0.00† | 0.64 | 0.00† | 0 | 0.40 |
|  | **95% UCL** | NA | 0.13 | NA | NA | NA | 13.14 | 0 | 3.90 | 3.42 | 1.65 | 0 | 4.24 |
| **Warm Deserts (n=31)** | **% Available** | 8.17 | 72.84 | 0.20 | 3.32 | 1.63 | 0.74 | 6.03 | 2.74 | 1.11 | 0.78 | 0.45 | 1.99 |
|  | **% Used** | 6.32 | 0.33 | 12.70 | 3.48 | 10.10 | 6.77 | 3.26 | 11.81 | 3.23 | 5.25 | 0 | 36.75 |
|  | **w_i_** | 0.77 | 0.005** | 63.19 | 1.05 | 6.20 | 9.21 | 0.54 | 4.30 | 2.90 | 6.76 | 0 | 18.46* |
|  | **SE** | 0.23 | 0.002 | 22.21 | 0.73 | 3.13 | 6.52 | 0.39 | 1.46 | 1.03 | 4.52 | 0 | 4.79 |
|  | **95% LCL** | 0.10 | 0.00† | 0.00† | 0.00† | 0.00† | 0.00† | 0.00† | 0.13 | 0.00† | 0.00† | 0 | 4.73 |
|  | **95% UCL** | 1.44 | 0.01 | 126.81 | 3.15 | 15.16 | 27.90 | 1.66 | 8.48 | 5.86 | 19.70 | 0 | 32.19 |
